# Phylogenetic Relationships and Species Delimitation in *Pinus* Section *Trifoliae* Inferrred from Plastid DNA

**DOI:** 10.1371/journal.pone.0070501

**Published:** 2013-07-30

**Authors:** Sergio Hernández-León, David S. Gernandt, Jorge A. Pérez de la Rosa, Lev Jardón-Barbolla

**Affiliations:** 1 Departamento de Botánica, Instituto de Biología, Universidad Nacional Autónoma de México, México City, Mexico; 2 Instituto de Botánica, Universidad de Guadalajara, Zapopan, Jalisco, Mexico; 3 Departamento de Ecología Evolutiva, Instituto de Ecología, Universidad Nacional Autónoma de México, México City, Mexico; Consiglio Nazionale delle Ricerche (CNR), Italy

## Abstract

Recent diversification followed by secondary contact and hybridization may explain complex patterns of intra- and interspecific morphological and genetic variation in the North American hard pines (*Pinus* section *Trifoliae*), a group of approximately 49 tree species distributed in North and Central America and the Caribbean islands. We concatenated five plastid DNA markers for an average of 3.9 individuals per putative species and assessed the suitability of the five regions as DNA bar codes for species identification, species delimitation, and phylogenetic reconstruction. The *ycf1* gene accounted for the greatest proportion of the alignment (46.9%), the greatest proportion of variable sites (74.9%), and the most unique sequences (75 haplotypes). Phylogenetic analysis recovered clades corresponding to subsections *Australes*, *Contortae*, and *Ponderosae*. Sequences for 23 of the 49 species were monophyletic and sequences for another 9 species were paraphyletic. Morphologically similar species within subsections usually grouped together, but there were exceptions consistent with incomplete lineage sorting or introgression. Bayesian relaxed molecular clock analyses indicated that all three subsections diversified relatively recently during the Miocene. The general mixed Yule-coalescent method gave a mixed model estimate of only 22 or 23 evolutionary entities for the plastid sequences, which corresponds to less than half the 49 species recognized based on morphological species assignments. Including more unique haplotypes per species may result in higher estimates, but low mutation rates, recent diversification, and large effective population sizes may limit the effectiveness of this method to detect evolutionary entities.

## Introduction

Introgression, incomplete lineage sorting, gene duplication, and lateral gene transfer can result in discordance between gene genealogies and species trees [1,2,3,4,5]. The genus *Pinus* L. has provided several examples of introgression and incomplete lineage sorting [6,7,8,9,10,11]. Plastid DNA trees of pines are discordant with aspects of nuclear [11,12] and mitochondrial [10] DNA trees, resulting in conflicting relationships among the principal lineages (particularly at the taxonomic level of subsection), and among more closely related species [9]. Introgression and incomplete lineage sorting are favored by pine life history, which is characterized by wind pollination, weak reproductive isolating barriers, longevity, overlapping generations, and large effective population sizes.

Analysis of multilocus datasets has been advocated for phylogenetic inference and delimitation of species; however, the nuclear genome is the main source of multiple unlinked loci, and the biparental inheritance and diploid (or polyploid) nature of nuclear alleles result in slower coalescence times relative to organellar DNA, requiring more generations before alleles are monophyletic within a species [5]. Plastid DNA has played an important role in investigating phylogenetics and species limits in plants because it is easy to amplify and sequence; it is also predominantly uniparentally inherited, and evidently undergoes little to no recombination, resulting in a conservative size, structure, and gene order [13]. Its uniparental mode of inheritance results in faster coalescence times than nuclear DNA but also makes it highly susceptible to "plastid capture" via interspecific gene flow [2]. Nevertheless, organellar DNA from multiple individuals per species can be used with varying accuracy for species identification as “DNA barcodes” [14] and for inferring species phylogeny. These data can also be used to delimit species [15,16,17,18,19,20,21,22], although multiple independent sources of data are preferable, particularly when introgression and incomplete lineage sorting are suspected.

*Pinus* section *Trifoliae* (*Pinus* subgenus *Pinus*), the "North American hard pines", are medium to large trees native to North and Central America and the Caribbean islands [23]. The section is divided into three subsections, *Contortae*, *Australes*, and *Ponderosae* [24], and includes several of the world’s most ecologically and economically important tree species, including *P* *. contorta* (lodgepole pine), *P* *. ponderosa* (ponderosa pine), *P* *. caribaea* (Caribbean pine), *P* *. radiata* (radiata pine), and *P* *. taeda* (loblolly pine) [25]. Taxonomic classifications based on morphological criteria (and often supported by crossability and differences in secondary metabolite profiles) are well advanced in pines, but they disagree somewhat among different workers. Price et al. [26] recognized 47 species in the section, Eckenwalder [27] recognized 44, and Farjon [28] recognized 45. The species of section *Trifoliae* once were classified together with Eurasian hard pines [29,30,31], but now are recognized as a natural group based on phylogenetic studies of plastid DNA [24,26,32,33,34]. The morphology-based circumscription of subsection *Contortae* was corroborated by plastid phylogenies, but not with nuclear ribosomal DNA [12]. The morphological characters that distinguish between subsections *Ponderosae* and *Australes* remain unclear, and traditional circumscriptions of the subsections were changed for several Mexican species based on their position in plastid DNA trees.

Nearly complete plastome sequences have been used to infer a robust gene tree for most species of *Pinus*, including 39 of the approximately 49 species of section *Trifoliae* [34]. Nevertheless, more thorough species and population sampling is needed to understand phylogenetic relationships and to evaluate how well species delimitations based primarily on morphology coincide with plastid DNA lineages. Two earlier phylogenetic studies included multiple individuals per species in *Pinus* subsection *Ponderosae*, revealing species-level genealogical nonmonophyly for two nuclear loci and for plastid sequences [11,35]. Failure of multiple DNA markers to form species-specific clades is consistent with a time lag during speciation between morphological divergence (e.g., of leaves and seed cones, which presumably are subject to natural selection), and reciprocal monophyly of DNA markers [36]. In pines, incongruence between species limits and gene trees can be attributed to introgression and incomplete lineage sorting, but may be exacerbated by inadequate species concepts and low levels of molecular variation. This incongruence represents an important challenge to DNA bar coding and DNA-based species delimitation, and is an interesting aspect of systematics and evolution.

Here we present a DNA sequence alignment of five plastid loci for multiple individuals of all recognized species in *Pinus* section *Trifoliae*. Two of the loci (*matK* and *rbcL*), were selected as the “core” DNA bar codes for land plants and a third (the *trnH-psbA* intergenic spacer) was recommended as a supplementary locus [14]. The other two (*ycf1* and the *trnD-trnY-trnE* intergenic spacer region) were selected based on their high variation in an alignment of the first two plastomes available for pines, those of *P* *. thunbergii* and *P* *. koraiensis* [35]. The objectives of this study are to (i) evaluate variability in the five loci, and their ability to discriminate pine plastid haplotypes, (ii) infer plastid DNA relationships for multiple individuals of all recognized species in *Pinus* section *Trifoliae*, (iii) quantify how many species have haplotypes that form either monophyletic or paraphyletic groups, and (iv) use the general mixed Yule-coalescent (GMYC) method to estimate the number of plastid lineages and compare those estimates to the number of species recognized based on morphology.

## Materials and Methods

**Figure 1:**
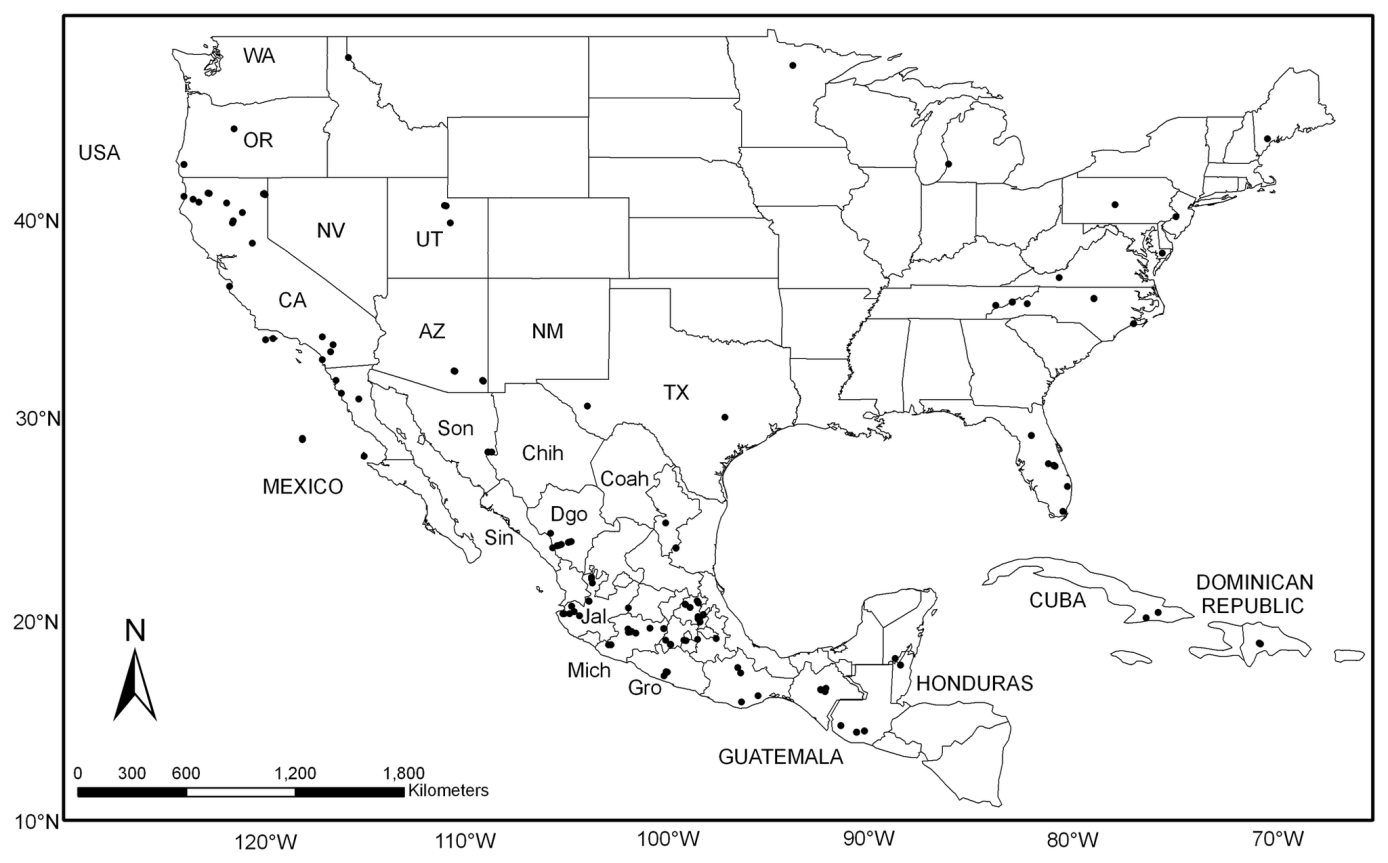
Geographic distribution of individual samples of *Pinus* section *Trifoliae*. Three of the 191 individuals are of unknown provenance.

Field collections of two to three branches, usually with seed cones, were made throughout the range of *Pinus* section *Trifoliae* (Figure 1). These were complemented with botanical garden and arboretum collections, including several of unknown provenance and two artificial hybrids; vouchers were deposited in herbaria (Appendix S1). Collections in Mexico were conducted under the successive permits SPGA/DGVS/00929, SGPA/DGGFS/712/1502/09, and SGPA/DGGFS/712/3692/10 issued to DSG by the Secretaria de Medio Ambiente y Recursos Naturales. No specific permits were required for material collected in Cuba, the Dominican Republic, Guatemala, and the United States; the locations are not privately-owned or protected in any way and none of the species collected in the field are endangered.

Here we followed the treatment of Farjon [28] for our hypothesis of species delimitation with four exceptions: 1) the recently described *Pinus* *georginae* [37] was treated as distinct from *P* *. praetermissa* , 2) *P* *. scopulorum* was treated as distinct from *P* *. ponderosa* rather than as a variety, 3) *P* *. chihuahuana* was treated as distinct from *P* *. leiophylla* , and 4) *P* *. yecorensis* was treated as distinct from *P* *. pseudostrobus* (Appendix S2). Recognition of these species is supported by morphological differences and by divergence in plastid DNA (see below). Several species recognized preliminarily in a previous plastid study of *Pinus* subsection *Ponderosae* [35] were reclassified following Farjon [28], mainly because their species status is not widely accepted by taxonomists and because we were unable to differentiate their plastid haplotypes from other species. These reclassifications were as follows: *P* *. cooperi* was treated as *P* *. arizonica* var. *cooperi* , *P* *. donnell-smithii* as *P* *. hartwegii* , *P* *. nubicola* as *P* *. pseudostrobus* var. *apulcensis* , and *P* *. washoensis* as *P* *. ponderosa* . Two to fourteen individuals for each of the 49 species were selected for characterization, and all species except *P* *. yecorensis* were represented by at least two populations.

Total genomic DNA was extracted from leaves with the CTAB method [38]. We sequenced fragments of the coding regions maturase K (*matK*), ribulose-1,5-bisphosphate carboxylase oxygenase (*rbcL*), and *ycf1*; the latter is an open reading frame of unknown function. We also sequenced the intergenic spacer between *trnH* and *psbA* (*trnH-psbA*) and the spacer regions spanning *trnD* and *trnY* (partial), and *trnY* and *trnE* ("*trnD-trnY-trnE*"). The *trnD*-*trnY*-*trnE* marker also included 350 bp flanking *trnE* that was annotated as an open reading frame of unknown function (orf126) in the GenBank record of *P* *. thunbergii* (NC_001631). PCR and sequencing protocols were the same as described previously [35] except that additional primers and plastid DNA regions were used (Appendix S3) [39,40,41,42,43]. New primers for *ycf1* were designed with Primer3 [44]. *Pinus* section *Trifoliae* has consistently been monophyletic and sister to *Pinus* section *Pinus* in previous plastid DNA studies [24,32]. We included two species from the latter section ( *P* *. resinosa* from northeastern North America and *P* *. thunbergii* from east Asia) as outgroups. *Pinus* *resinosa* was from a recent collection, while the plastome sequence of *Pinus* *thunbergii* was downloaded from GenBank (NC_001631 [45]).

Sequences were assembled and edited in Sequencher ver. 4.8 (Gene Codes, Inc., Ann Arbor, Michigan), imported into BioEdit [46], and aligned with MAFFT 6 [47]. The matrix had 193 terminals including the two outgroups. *Pinus* section *Trifoliae* was represented by a mean of 3.9 individuals per species. The matrix had 0.82% missing data (0-14.7% for individual terminals). The *trnH-psbA* spacer was the least complete, mainly because of difficulty amplifying and sequencing in subsection *Contortae* (Table 1), which has two copies of the *psbA* gene [48]; we used unidirectional reads of the *trnH* primer for the four species in this section, resulting in 170-193 bp of these sequences adjacent to the *psbA* end coded as missing ("N"). Sequences were deposited in Barcode of Life Datasystems (BOLD) [49] and GenBank, and the alignment was deposited in TreeBase (Study accession URL: http://purl.org/phylo/treebase/phylows/study/TB2:S13570).

**Table 1:** Characteristics of the plastid DNA sequence alignment for *Pinus* section *Trifoliae*.

**Parameter**	**Group**	***matK***	***rbcL***	***ycf1***	***trnD-trnY-trnE***	***trnH-psbA***
Number of sequences						
	subsect. *Australes*	93	95	95	95	95
	subsect. *Contortae*	10	10	10	10	10
	subsect. *Ponderosae*	85	86	86	86	86
	Aligned length	812	607	2541	834	631
Length range						
	subsect. *Australes*	812	607	2451-2484	757-759	612-618
	subsect. *Contortae*	812	607	2430	758	*
	subsect. *Ponderosae*	812	607	2451	754-760	617
G + C content						
	subsect. *Australes*	0.363	0.440	0.365	0.372	0.381
	subsect. *Contortae*	0.361	0.437	0.365	0.378	0.378
	subsect. *Ponderosae*	0.362	0.442	0.363	0.375	0.380
Number of variable but parsimony-uninformative sites						
	subsect. *Australes*	0	0	10	0	1
	subsect. *Contortae*	0	0	10	1	2
	subsect. *Ponderosae*	3	0	9	3	0
	sect. *Trifoliae*	3	0	16	4	1
Number of informative sites						
	subsect. *Australes*	3	2	81	11	7
	subsect. *Contortae*	0	3	35	2	3
	subsect. *Ponderosae*	3	0	28	5	5
	sect. *Trifoliae*	7	11	184	24	17
W-Theta per site						
	subsect. *Australes*	0.00073	0.00070	0.00712	0.00259	0.00235
	subsect. *Contortae*	0	0.00175	0.00641	0.00140	0.00177
	subsect. *Ponderosae*	0.00150	0	0.00292	0.00211	0.00164
Number of haplotypes						
	subsect. *Australes*	4	3	43	13	10
	subsect. *Contortae*	1	3	8	3	4
	subsect. *Ponderosae*	7	1	25	8	6
	sect. *Trifoliae*	11	7	75	24	18

* The *trnH-psbA* sequences are incomplete for *Pinus* subsection *Contortae*.

Molecular variation was measured with DnaSP version 5 [50], and PAUP* version 4.0b10 [51]. Parsimony searches were performed in PAUP* with and without simple indel coding [52] as implemented with SeqState version 1.4.1 [53]. For parsimony, heuristic searches employed 1000 ratchet iterations [54] using a batch file generated with PRAP2 [55] that assigned a differential weight of 2 to 25% of the characters. Nucleotide substitution models were chosen in JModeltest [56,57]. Eighty-one models for maximum likelihood optimized trees were chosen for five data partition configurations: 1) no partitions, 2) partitions of the plastid regions into three subsets (spacer, *ycf1*, and *matK* + *rbcL*; the latter two genes evolve more slowly than *ycf1*), 3) partitions into five subsets corresponding to the five plastid regions, 4) partitions of the five regions plus a partition in *ycf1* between the first and second codon position and the third position, and 5) partitions of each of the five regions, with separate partitions for the first, second, and third position of *ycf1*. Maximum likelihood analysis was performed in GARLI version 2.0 [58] using 50 replicates of stepwise addition, 50 attachments per taxon, and a termination threshold of 5,000 generations without a score improvement of 0.001. Branch support was measured for the parsimony and likelihood trees using 1000 (100 random addition sequence, saving ten trees per replicate) and 100 nonparametric bootstrap replicates, respectively [59]. We counted the number of (hypothesized) species recovered as monophyletic or paraphyletic, and the number of species that did not share haplotypes with other species (having diagnostic or exclusive haplotypes).

Ultrametric trees and the absolute age of lineages were estimated with a relaxed molecular clock using parameters specified in BEAUti version 1.71 and implemented in BEAST version 1.7.1 [60]. The fossil record of pinaceous wood and ovulate cones is imperfectly understood, but an early Cretaceous age for the genus has been widely accepted [61,62,63]. We used two calibration combinations; the first was a secondary calibration based on results from a previous molecular clock study of nuclear DNA that assumed a late Cretaceous divergence (85 million years ago; ma) of the two *Pinus* subgenera [64], and gave divergence time estimates for the most recent common ancestor (MRCA) of section *Trifoliae* and of the MRCA of subsections *Australes* and *Ponderosae* of 18 ma and 15 ma, respectively [65]. These results were supported by a separate plastid DNA study using different calibration points outside of the *Pinus* stem group that estimated a 17 or 20 ma age for the MRCA of section *Trifoliae* [66].

For the second calibration we added one point based on fossils interpreted as crown members of subsection *Australes*. Three fossil species described from the Miocene and Pliocene (no later than 5.33 ma [67]) of California, *P* *. lawsoniana* Axelrod, *P* *. pretuberculata* Axelrod, and *P* *. masonii* Dorf, resemble the California closed-cone pines *P* *. radiata* , *P* *. attenuata* , and *P* *. muricata* , respectively [68]. Acceptance of the hypothesized phylogenetic position and age of these fossils could increase the estimated molecular clock based age of *Pinus*. The fossil record of the California closed-cone pines may be even older, because *P* *. pretuberculata* as circumscribed by Axelrod also includes material as old as 12 ma.

Only unique plastid haplotypes were included in the molecular clock analysis. Identical haplotypes were identified for removal by examining pairwise distances in PAUP*. Preliminary runs with seven data subsets, one for each locus, and with additional partitions specified for the first, second, and third codon position of *ycf1*, gave effective sample sizes less than 200 for several parameters, including the tree prior, suggesting insufficient convergence. Therefore, we reduced the number of data subsets to three, one for the spacers (*trnH*-*psbA* and *trnD-trnY-trnE*), one for *ycf1*, and one for the two slower evolving genes (*matK* and *rbcL*). The Bayesian Information Criterion (BIC) was applied in jModeltest to guide the choice of either HKY or GTR nucleotide substitution models depending on which model accounted for all the parameters for each data subset and therefore represented the best model approximation available in the BEAUti interface. We also included the rate heterogeneity parameter (G), but not invariant sites (I), because the latter failed to converge in preliminary runs. We designated an uncorrelated log normal relaxed molecular clock with a Yule speciation tree prior ranging from 0 to 1x10^100^ and a uniform ucld.mean prior ranging from 0 to 1 [69,70]. An 18 ± 2 s.d. ma normal prior was specified for the split between subsections *Contortae* and *Australes* + *Ponderosae*, and a 15 ± 2 s.d. ma prior for the split between subsections *Australes* and *Ponderosae*. Five independent Markov chains of 40,000,000 generations were run, saving the results every 4000th generation. The runs were examined for convergence in Tracer version 1.5, and the maximum clade credibility tree with mean node heights was generated from the combined runs after eliminating 10% of the trees for burnin in TreeAnotator version 1.7.2.

We used the maximum clade credibility chronograms from the relaxed molecular clock analyses to determine whether an increase in branching rates could be observed in a lineage-through-time plot [71] indicative of a transition from (Yule) speciation to coalescent processes (a species-coalescent boundary), estimate the number of entities that may correspond to species using the general mixed Yule-coalescent (GMYC) method, and compare the number of entities to the number of species recognized based on morphological criteria. The trees were read into the ape [72] and splits [73] package of R version 2.15.1 [74], the two outgroups were pruned, and GMYC models were tested against the null hypothesis of a single coalescent branching rate. The number of entities (as clusters and singletons) was estimated by applying GMYC for single and multiple thresholds, and using a multimodel Akaike information criterion with a model cutoff of delta AICc = 7 [18,21,22].

## Results

### Plastid DNA variation

The concatenated matrix was 5,425 bp in length, representing 4.4% of the *Pinus* plastome based on the complete sequence of *P* *. thunbergii* . All individuals of *P* *. jeffreyi* had a ten bp inversion in the *trnH-psbA* spacer. Exclusion of the inversion resulted in an alignment with 91 variable but parsimony-uninformative sites and 368 parsimony-informative sites. Deleting the two outgroup sequences resulted in 23 variable and 243 parsimony-informative sites and 95 unique haplotypes (Table 1). A total of 27 indels were inferred, ten in the *trnH-psbA* spacer, eight in the *trnD-trnY-trnE* spacer region, and nine in *ycf1*. The region annotated as “ORF128” adjacent to the *trnD-trnY-trnE* spacer of *P* *. thunbergii* was disrupted by a mononucleotide A/T repeat that varied in length from 8 to 11 bp in subsections *Ponderosae* and *Australes*. The mononucleotide repeat was 6 bp in subsection *Contortae* and 7 bp in the two outgroup sequences.

**Figure 2:**
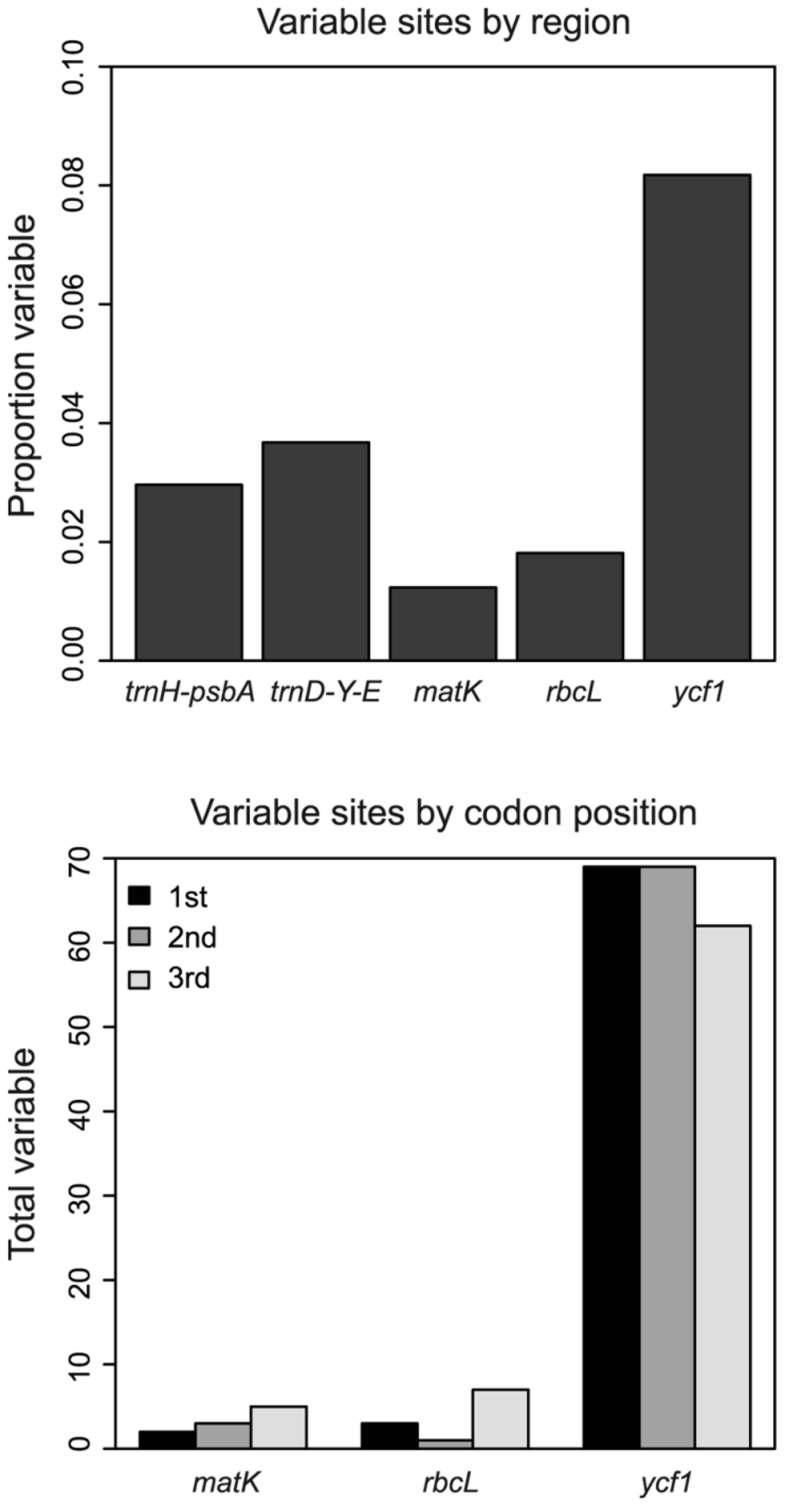
Variation of the plastid markers in *Pinus* section *Trifoliae*. Above: The proportion of variable sites for each of the five markers. Below: the total number of variable sites per codon position for the three protein coding regions.

The *ycf1* gene accounted for 46.9% of the alignment and exceeded all other regions in variation, with 74.9% of the variable and informative sites (16 variable and 184 parsimony-informative) in section *Trifoliae*. It had the highest average nucleotide diversity per site (π) of 0.0112; in comparison, π was 0.00495 for *trnD-trnY-trnE*, the second most variable region, and was as low as 0.00118 for *matK*. The latter region had only 10 variable sites in section *Trifoliae*, 7 of which were parsimony-informative. Variation was comparably low in *rbcL*, with 11 variable sites, all of which were parsimony-informative (π = 0.00375). For the three coding regions, substitutions were most frequent in the third codon position for *matK* and *rbcL*, but in *ycf1*, substitutions in the first and second codon position exceeded those in the third (Figure 2). The *ycf1* gene was the only region to yield more haplotypes (75) than recognized species. Subsections *Australes* and *Ponderosae* shared one *matK* haplotype and two *trnH-psbA* haplotypes. In contrast, *rbcL*, *ycf1*, and *trnD-trnY-trnE* could all be used to unambiguously assign individuals to a *Pinus* subsection. Concatenating the *matK* and *rbcL* sequences as recommended for use as DNA bar codes yielded 16 haplotypes and permitted the discrimination of four of the 49 (8%) section *Trifoliae* species.

The number of variable sites was 2.2 times greater in subsection *Australes* (115 variable and parsimony informative sites) than in subsection *Contortae* (56 variable sites) and subsection *Ponderosae* (56 variable sites). Subsection *Australes* also had the most *ycf1*, *trnD-trnY-trnE*, and *trnH-psbA* haplotypes (Table 1).

### Phylogenetic relationships

The heuristic search of the alignment with gaps treated as missing recovered 28 most parsimonious trees (MPTs) of length (L) = 606 steps, consistency index (CI) = 0.828, consistency index excluding parsimony uninformative characters (CIexc) = 0.792, and retention index (RI) = 0.979 (not shown). Including 27 gaps using simple indel coding resulted in 394 parsimony informative and 98 variable but uninformative characters, and the heuristic search recovered 247 unique MPTs (Appendix S4; L = 648, CI = 0.826, CIexc = 0.789, RI = 0.978).

**Figure 3:**
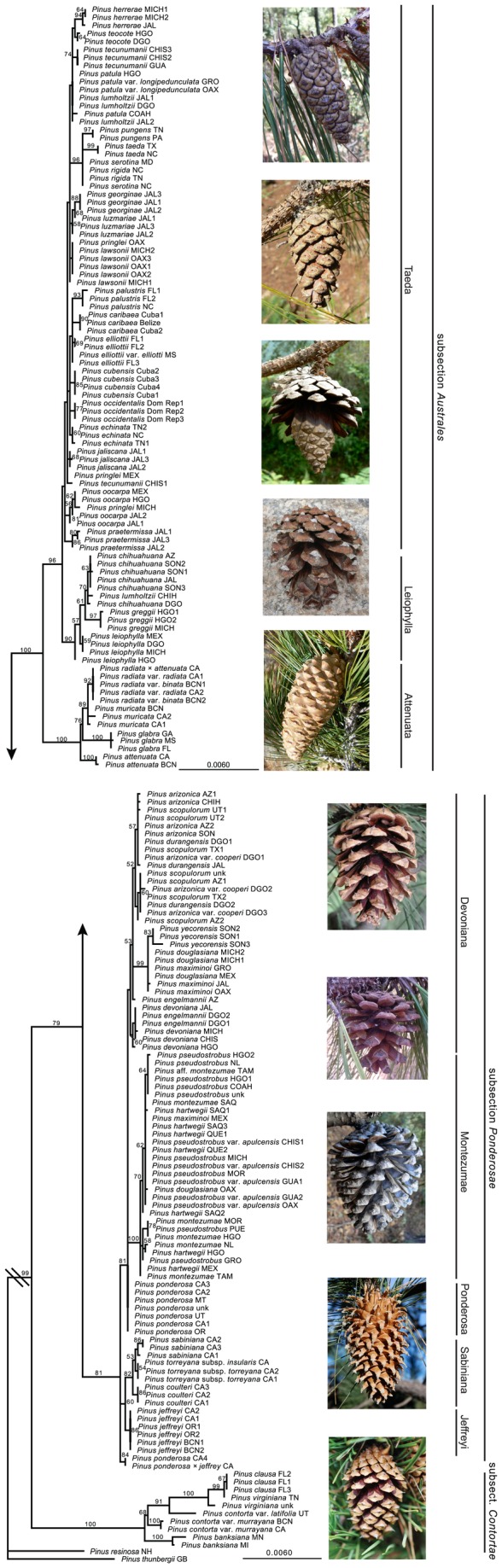
Maximum likelihood tree. Bootstrap values greater than 50% are shown at branches. A. *Pinus* subsection *Australes*. Images from top to bottom are of *P* *. lumholtzii* , *P* *. herrerae* , *P* *. oocarpa* , *P* *. chihuahuana* , and *P* *. attenuata* . B. *Pinus* subsections *Ponderosae* and *Contortae*. Images from top to bottom are *P* *. scopulorum* , *P* *. douglasiana* , *P* *. pseudostrobus* var. *apulcensis* , *P* *. coulteri* , and *P* *. contorta* . The branch leading to the outgroups has been truncated.

The partitioning strategy that divided the data matrix into five subsets, one for both spacer regions, one for *matK* + *rbcL*, and three for the three codon positions of *ycf1*, recovered the best likelihood tree and gave the lowest AIC value (Appendix S5 Figure 3). The optimal tree for the five subset partition was found in one of 50 heuristic search replicates and agreed in all main relationships with the parsimony strict consensus, although the likelihood tree was better resolved near the tips. The best models for all the partitions included either a parameter for proportion of invariant sites (I), for rate variation among sites (G), or both.

The three subsections, *Australes*, *Contortae*, and *Ponderosae*, were recovered as monophyletic with high support, with subsection *Contortae* sister to subsection *Australes* + *Ponderosae*. In the subsection *Australes* clade, the California closed-cone pines, sometimes classified separately as subsection *Attenuatae* (indicated as “ *Attenuata* ” in Fig. 3a), formed a well-supported clade, but nested within it were three individuals of *P* *. glabra* from the southeastern U.S; the *Attenuata* clade was sister to the rest of subsection *Australes*. Another well-supported clade (labeled as “Leiophylla” in Fig. 3a) included *P* *. greggii* , *P* *. chihuahuana* , *P* *. leiophylla* , and a single *P* *. lumholtzii* individual. The remaining sequences formed a poorly supported clade (“Taeda”). Other groups of subsection *Australes* sequences that received bootstrap support greater than 70% included *P* *. cubensis* (including individuals corresponding to " *P* *. maestrensis* " here considered as a synonym of *P* *. cubensis* ), *P* *. georginae* (a recently described species), *P* *. palustris* , *P* *. caribaea* , *P* *. occidentalis* , *P* *. praetermissa* , *P* *. tecunumanii* (three of four individuals), and a clade of all individuals of the southeastern U.S. species *P* *. rigida* , *P* *. serotina* , *P* *. pungens* , and *P* *. taeda* .

In subsection *Ponderosae* (Fig. 3b), a "Sabiniana" clade included *P* *. coulteri* sister to *P* *. torreyana* + *P* *. sabiniana* . Six *P* *. jeffreyi* individuals were sister to this clade. Identical sequences from two individuals of *P* *. ponderosa* from California were in an unresolved trichotomy with the Sabiniana + Jeffreyi clade and a clade of the remaining subsection *Ponderosae* sequences. Another seven identical *P* *. ponderosa* sequences from more northern coastal and interior localities occurred in a trichotomy with a " *Devoniana* " and a “Montezumae” clade. The *Devoniana* clade included southwestern U.S. and northern Mexico taxa *P* *. scopulorum* and *P* *. arizonica* vars. *arizonica* and *cooperi*, and the Mexican and Central, American species *P* *. devoniana* , *P* *. durangensis* , *P* *. engelmannii* , *P* *. maximinoi* , *P* *. douglasiana* , and *P* *. yecorensis* . The Montezumae clade comprised species with distributions in Mexico and Central America ( *P* *. pseudostrobus* , *P* *. montezumae* , *P* *. hartwegii* , and a single individual each of *P* *. maximinoi* , and *P* *. douglasiana* ); an individual from the Sierra Madre Oriental in northeast Mexico with unusual combinations of morphological characters, tentatively identified as P. aff. *montezumae*, was in a clade with *P* *. pseudostrobus* and *P* *. montezumae* , also from the Sierra Madre Oriental.

In subsection *Contortae*, sequences from two *P* *. banksiana* individuals were monophyletic and sister to a clade of the remaining three species. Two *P* *. contorta* var. *murrayana* sequences formed a clade that was successively paraphyletic to a *P* *. contorta* var. *latifolia* sequence and two *P* *. virginiana* individuals that were in turn paraphyletic to a clade of three *P* *. clausa* individuals.

### Plastid DNA based species delimitation

Allelic monophyly was observed for 23 of the 49 recognized species (47%; Table 2), and paraphily was observed for another nine species. In subsection *Contortae*, haplotypes for two species ( *P* *. banksiana* and *P* *. clausa* ) formed monophyletic groups. Alleles were monophyletic for five of the 16 species of subsection *Ponderosae* ( *P* *. coulteri* , *P* *. jeffreyi* , *P* *. sabiniana* , *P* *. torreyana* , and *P* *. yecorensis* ) and for 16 of the 29 species of subsection *Australes*. Sequences were paraphyletic for the other two species of subsection *Contortae* and for another seven species of subsection *Australes*. Of the remaining 17 species with polyphyletic sequences (six in subsection *Australes* and eleven in subsection *Ponderosae*), two were considered diagnosable because all of their haplotypes were unique compared to those of all other species. One of these species was *P* *. ponderosa* , which had biphyletic but unique haplotypes. Two caveats applied in this case, first, it depended on our assignment of most morphologically similar interior populations to *P* *. scopulorum* , and second, one individual with a *P* *. ponderosa* haplotype was an artificial hybrid with *P* *. jeffreyi* . In subsection *Australes*, sequences of *P* *. tecunumanii* were also polyphyletic but diagnostic. The remaining 15 species had at least one haplotype that was identical to a haplotype from another species.

**Table 2:** Comparison of the number of morphological species with plastid DNA haplotypes and their relationships.

**Subsection**	**Individuals/species**	**Number of haplotypes**	**Monophyletic Species (%)**	**Diagnosable Species (%)**
*Australes*	95/29	51	16 (55%)	24 (83%)
*Contortae*	10/4	8	2 (50%)	4 (100%)
*Ponderosae*	86/16	34	5 (31%)	6 (38%)
TOTAL	191/49	93	23 (47%)	34 (69%)

The number of diagnosable species is the sum of all putative species with monophyletic, paraphyletic, and polyphyletic but unique haplotypes.

### Molecular Dating and Lineage Delimitation Using GMYC

**Figure 4:**
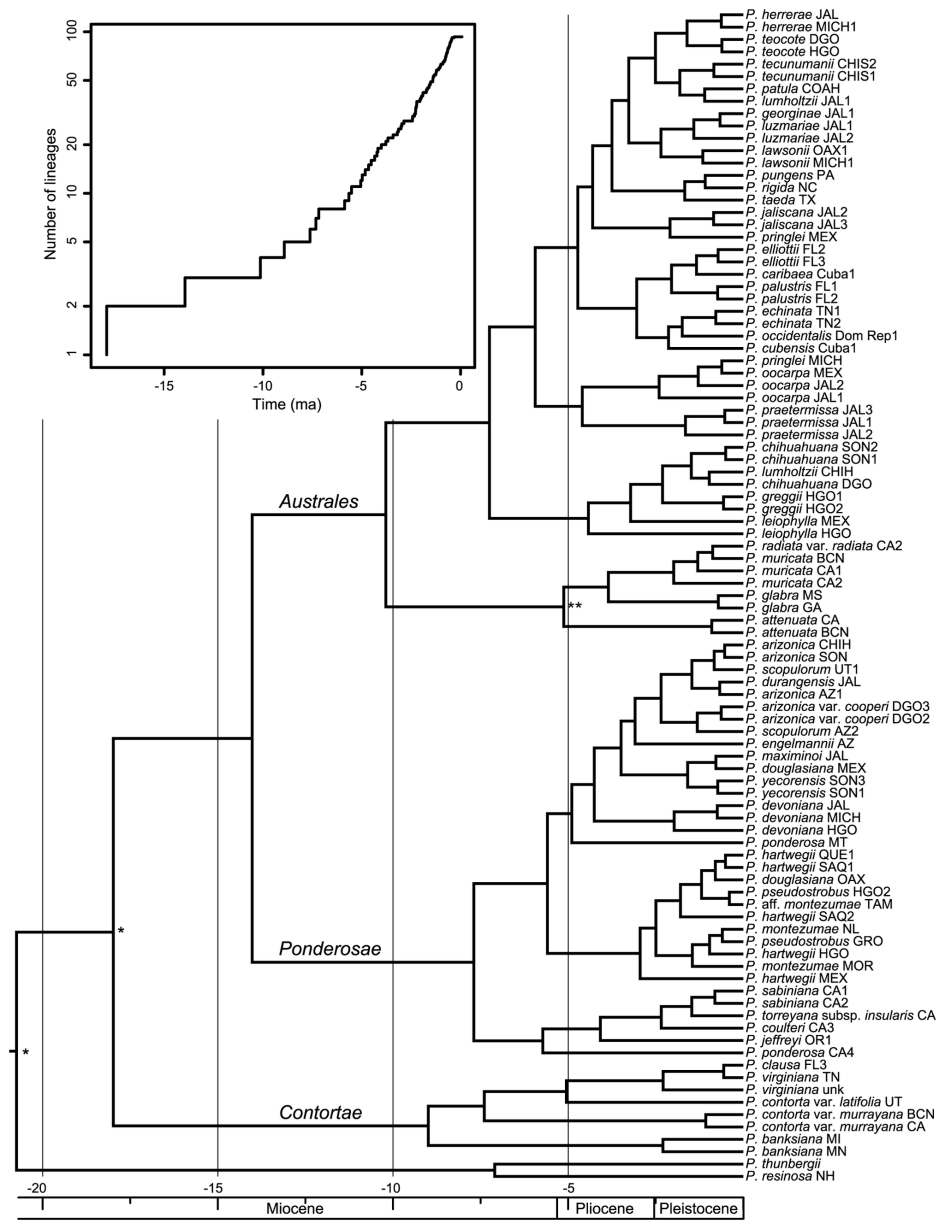
Chronogram for *Pinus* section *Trifoliae* using three calibration points and lineage-through-time plot. Chronogram for section *Trifoliae*. The outgroup has been removed. The two secondary calibration points inferred from a previous relaxed molecular clock are indicated with an asterisk (*), and a fossil calibration point attributed to the California closed-cone pines is indicated with a double asterisk (**). The corresponding lineage-through-time plot is given on the upper left. The transition from speciation to coalescence branching processes gave nonsignificant test results.

The relaxed molecular clock derived from the 95 unique sequences and 5415 sites and using only the secondary calibration of the MRCA of section *Trifoliae* and of subsections *Australes*-*Ponderosae* resulted in a crown group divergence for section *Trifoliae* of 18.0 ma with a 95% highest posterior density (HPD) of 14.7–21.4, a 14.0 ma (95% HPD 11.0–16.0) age for the MRCA of *Australes*-*Ponderosae*, and crown group ages of 8.8 (95% HPD 4.8–13.6), 10.3 (95% HPD 6.8–13.8), and 7.7 ma (95% HPD 4.3–11.6) for subsections *Contortae*, *Australes*, and *Ponderosae*, respectively (Appendix S6). Inclusion of the fossil calibration point for the *Attenuata* clade (Figure 3) gave the same age estimates for section *Trifoliae* (18.0 ma; 95% HPD 14.8–21.4; Figure 4), and for the MRCA of *Australes*-*Ponderosae* (14.0 ma; 95% HPD 11.0–17.0). This also resulted in ages of the same three subsectional crown nodes of 9.0 (95% HPD 4.9–13.5), 10.2 (95% HPD 6.9–13.7), and 7.7 ma (95% HPD 4.3–11.4; Figure 4).

The trees resulting from both calibrations gave similar GMYC results (Appendix S7). Here we report the statistics for the tree that included both the secondary and fossil calibration points. The lineage-through-time plot did not show a sudden increase in slope indicative of a transition from (Yule) speciation branching rates to coalescent branching rates (Figure 4). The single threshold model did not result in a significant improvement in likelihood over the null coalescent model (likelihood ratio = 3.17, test statistic = 0.21, not significant). Nevertheless, it gave a threshold time estimate of 2.51 ma, resulting in 20 clusters (C.I. estimated as two log-likelihood units from the maximum likelihood solution = 1–25), and an additional eight lineages consisting of single sequences, giving a total of 28 entities (C.I. = 1−36). Comparison of the multiple to the single threshold method gave a likelihood ratio of 10.08 (test statistic = 0.0065, significant). The multiple method gave threshold times of 7.69, 3.22, and 2.05 ma, resulting in 14 clusters (C.I. = 10−20) and 18 entities (C.I. = 12−27). Application of multimodel AICc (delta AICc = 7; 76 best of 142 total models) yielded an estimate of 14.27 clusters (var. = 4.84) and 22.92 entities (var. = 9.19).

We also examined the cluster probabilities (the probability that two tips of the tree belong to the same cluster) assigned to the tree nodes based on the multimodel AICc. A probability threshold of 0.95 identified a total of 34 entities, four for subsection *Contortae* (three clusters and one singleton), 11 for subsection *Ponderosae* (5 clusters and 6 singletons), and 19 for subsection *Australes* (14 clusters and 5 singletons). Seventeen of these entities corresponded to a single species, ten corresponded to two species, six corresponded to three species, one corresponded to four species, and one corresponded to five species. Lowering the cluster probability threshold to 0.50 reduced the number of entities to 25.

## Discussion

### Prospects for plastid DNA bar codes of pines

DNA sequences offer great potential for providing fast and accurate species identifications [75], but the short *matK* and *rbcL* fragments chosen for plant DNA bar codes are known to have low substitution rates and thus low discriminatory power [14]. Low variation in the *matK* and *rbcL* of *Pinus* section *Trifoliae* was documented previously [24,35], but not specifically as DNA bar code markers. The *matK* and *rbcL* fragments corresponding to the DNA bar codes were the least variable of the five regions evaluated. Although *matK* and *rbcL* were variable enough to place an unknown sample to section *Trifoliae*, only the *rbcL* fragment was capable of placing all individuals to one of the three subsections. Both *matK* and *rbcL* were usually identical for closely related species and this was reflected in a low success rate for species discrimination. Evaluation of seven plastid markers across land plants reported an average of 72% species discrimination when using *matK* + *rbcL* in combination [14]. This same combination gives only 8% species discrimination in *Pinus* section *Trifoliae*, a decidedly poor performance for some of the world’s most ecologically and economically important trees.

The *trnH-psbA* and *trnD-trnY-trnE* spacers were of comparable length but more variable than the *matK* and *rbcL* fragments. However, they were not variable enough to discriminate most species within *Pinus* subsections, particularly in subsection *Ponderosae*. The *trnH-psbA* spacer can be amplified with universal primers designed originally for flowering plants, which makes it an attractive option for adding discriminatory power when using multiple loci for DNA bar coding taxonomically diverse floras. However, two *trnH-psbA* haplotypes were shared across subsections *Australes* and *Ponderosae* and we were unable to obtain bidirectional reads for subsection *Contortae*.

Only *ycf1* yielded on average more than one haplotype per species. The greater relative length of the sequenced fragment (ca. 2400 bp vs. 600–800 bp) was an important factor, but *ycf1* was also by far the most variable on a per-site basis (Figure 2), and therefore was more useful for discriminating plastid lineages, even when considering only a 600-800 bp fragment that can be sequenced with typical bidirectional Sanger sequencing reads. The greater variation and an excess of nonsynonymous compared to synonymous substitutions are consistent with positive selection acting on *ycf1* in *Pinus* [76]. Primers have been designed to amplify *ycf1* throughout Pinaceae [77], but these may not work in other conifer families, much less for more distantly related land plants. Therefore, although *ycf1* has already proved useful for identifying pine species [78], it does not fulfill the universality requirement of a DNA bar code. Nevertheless, if a sample is already known to belong to *Pinus* or Pinaceae (e.g., based on morphology or on an *rbcL* sequence) then genus or family specific PCR primers could be used to determine its *ycf1* haplotype, which in turn is a useful proxy for species identification.

### Phylogenetic relationships within *Pinus* section *Trifoliae*

Here we provide the first phylogenetic analysis of plastid DNA for North American hard pines that includes all recognized species and multiple individuals per species. Parsimony and maximum likelihood analyses of plastid DNA recovered three principal lineages of North American hard pines, subsections *Australes*, *Contortae*, and *Ponderosae*, in agreement with previous plastid studies with less taxonomic sampling [24,32,34,76,79]. Morphological synapomorphies are unknown for section *Trifoliae*, and although these subsections coincide in some respects with the influential classification of Little and Critchfield [31] based on morphology and crossability, emphasis on a limited subset of morphological characters resulted in a classification that conflicts with the plastid tree in several respects. If we take the classification of Little and Critchfield to illustrate this point, these authors did not recognize the species of section *Trifoliae* as a natural group, instead classifying hard pine species with deciduous fascicle sheaths, *P* *. leiophylla* , *P* *. chihuahuana* (as a variety of *P* *. leiophylla* ), and *P* *. lumholtzii* in a separate, morphologically heterogeneous section that also included some Eurasian pines. Subsection *Australes* as recognized here was classified by them into subsections *Australes* and *Oocarpae*, the former characterized by multinodal spring shoots, and mostly symmetrical cones, and the latter characterized by mostly oblique, serotinous cones. *Pinus* *lawsonii* and *P* *. teocote* , two species endemic to Mexico with symmetrical nonserotinous cones were classified by Little and Critchfield in subsection *Ponderosae* but are now classified in subsection *Australes* based on crossability data and DNA sequences. Finally, Little and Critchfield classified the "California big-cone pines" in subsection *Sabinianae*, separate from subsection *Ponderosae*. These differences are discussed more specifically below.

A similar plastid DNA data set for *Pinus* subsection *Ponderosae* was reported previously [35]. The main differences here are the use of a slightly longer and contiguous fragment of *ycf1*, an increase from 67 to 86 individuals, and the use of broader taxonomic concepts for four species. As with the earlier version of the plastid data, the three California big-coned pines, *P* *. coulteri* , *P* *. sabiniana* , and *P* *. torreyana* were monophyletic (the Sabiniana clade; Figure 3b). A close relationship between these species was recognized based on similarities in growth form, leaf, cone, and seed morphology, and their ability to form natural or artificial hybrids [80]. This group thus provides an example of congruence between plastid DNA relationships and other sources of evidence. Furthermore, both *P* *. coulteri* and *P* *. torreyana* can hybridize with *P* *. jeffreyi* , a species which also occurs in California, and both *P* *. jeffreyi* and *P* *. coulteri* can form hybrids with Californian populations of *P* *. ponderosa* . This genetic link between California big-coned pines and other members of subsection *Ponderosae* is reflected in the plastid tree, which recovers *P* *. jeffreyi* as the sister group to the California big-coned pines, and two Californian collections of *P* *. ponderosa* (one is actually a hybrid with *P* *. jeffeyi* ), in an unresolved trichotomy with all other species of subsection *Ponderosae*.

Ponderosa pine, or *P* *. ponderosa* , is one of the most taxonomically challenging taxa in subsection *Ponderosae*. In the broad sense, *P* *. ponderosa* also includes *P* *. scopulorum* as a variety ( *P* *. ponderosa* var. *scopulorum* Lemmon) [27,28,80]. Only minor, possibly clinal, morphological differences separate these taxa (e.g., *P* *. scopulorum* has higher proportions of two, rather than three leaves per fascicle, and *P* *. ponderosa* has longer leaves and larger ovulate cones and seeds). In contrast to their morphological similarity, sequences from *P* *. ponderosa* and *P* *. scopulorum* were polyphyletic, occurring as three divergent lineages both here and with nearly complete plastomes [34]. One of these lineages is distributed mainly in the southern Rocky Mountains and northern Mexico (“southern interior ponderosa pine” or *P* *. scopulorum* ), and is related to other taxa from the same geographic region like *P* *. arizonica* , which has also been synonymized with *P* *. ponderosa* by some workers [31]. The other two lineages correspond to “northern interior ponderosa pine” ( *P* *. ponderosa* in a restricted sense) and “Pacific coastal ponderosa pine” ( *P* *. ponderosa* var. *benthamiana* or *P* *. benthamiana* ) [34]. Our analysis included three individuals from the Rocky Mountains of Utah, but only two of these grouped with *P* *. scopulorum* , while the third was morphologically atypical (e.g., it had longer leaves), and grouped with northern *P* *. ponderosa* . Populations of ponderosa pine from Utah and Nevada are extremely variable in the proportion of needles in fascicles of three, typical of northern and Pacific *P* *. ponderosa* , and needles in fascicles of two, here treated as *P* *. scopulorum* [81]. This may be a result of hybridization, but more study is needed on these ecologically important and emblematic taxa to characterize their morphological and genetic variation.

Whereas we departed from recent taxonomic treatments [27,28] by recognizing *P* *. scopulorum* as separate from *P* *. ponderosa* , we followed them in treating *P* *. donnell-smithii* as a synonym of *P* *. hartwegii* and *P* *. nubicola* as a synonym of *P* *. pseudostrobus* var. *apulcensis* . Nevertheless, even with these broader species concepts, *P* *. hartwegii* , *P* *. pseudostrobus* , and *P* *. montezumae* had very low sequence divergence and shared haplotypes, consistent with previous studies reporting interspecific gene flow [7,82].

The broad circumscription of *Pinus* subsection *Australes* is based primarily on plastid DNA and to a lesser extent on the internal transcribed spacer of nuclear ribosomal DNA. In earlier classifications these species were divided into three subsections, *Attenuatae*, *Australes*, and *Oocarpae* [26,31], but none of these proposed groups was monophyletic in the plastid trees reported here or elsewhere [24,34,79]. *Pinus* subsection *Attenuatae* (“the California closed-cone pines”), was erected for *P* *. attenuata* , *P* *. muricata* , and *P* *. radiata* , the only three far western United States and Baja Californian species in this subsection, all with serotinous cones [26,83]. The three species formed a well-supported group, but included all three *P* *. glabra* sequences (Figure 3b). The latter species is also nested within the California closed-cone pines with nearly complete plastomes [34]. This species’ cones are non-serotinous, and it is geographically disjunct from the three Californian species, occurring in the southeastern United States. The position of *P* *. glabra* is one of the best possible examples of genealogical discordance of plastid DNA in *Pinus* section *Trifoliae*.

The ten species in subsection *Australes* as originally circumscribed by Little and Critchfield are distributed in eastern North America, the Caribbean, and Central America, and have symmetrical, non-serotinous cones and multinodal spring shoots. Four of these ( *P* *. pungens* , *P* *. rigida* , *P* *. serotina* , and *P* *. taeda* ) formed a well-supported clade, but nested among species of Little and Critchfield’s subsection *Oocarpae*. The placement of the other four species was not robust. Therefore, our results do no support the recognition of subsection *Oocarpae* but corroborate other molecular studies and the resulting subsectional classification of this group [24,34]. The nonmonophyly of Little and Critchfield’s subsections needs to be confirmed with independent data such as from another genomic compartment or morphology.

### Delimiting species with plastid DNA

Twenty-three of the 49 *Pinus* subsection *Trifoliae* species exhibited allelic monophyly, another nine were paraphyletic, two had polyphyletic but unique haplotypes, and 15 shared at least one plastid haplotype with another species. One explanation for shared haplotypes among species is that the morphological characters that distinguish these species are minor and insufficient to justify species status. For example, *P* *. serotina* is treated as a subspecies of *P* *. rigida* ( *P* *. rigida* subsp. *serotina* ) by Eckenwalder [27]. Here the four individuals sampled for these taxa (two each) had identical haplotypes. Also, *P* *. douglasiana* and *P* *. maximinoi* are recognized as distinct species in recent taxonomic treatments, but they are difficult to distinguish morphologically, and several of their sequences are identical. However, introgression is probably the most important cause of discordance between plastid DNA trees and species trees in plants [2]. Controlled crosses have thoroughly documented the weak intrinsic barriers to gene flow within *Pinus* subsections [80,84,85], and plastid DNA introgression in natural populations has been reported for all three subsections of North American hard pines [6,7,82,86,87]. Recent diversification via allopatric speciation followed by secondary contact may have promoted interspecific gene flow in this group. The range of intra- and interspecific morphological variation in species such as *P* *. arizonica* , *P* *. durangensis* , *P* *. ponderosa* , and *P* *. scopulorum* (subsection *Ponderosae*), and *P* *. tecunumanii* , *P* *. lawsonii* *,* *P* *. patula* , and *P* *. pringlei* (subsection *Australes*), is imperfectly understood, and natural hybridization is often suspected based on morphological intermediates. In these species, morphological and plastid DNA variation is relatively high, and cases of haplotype sharing occurs among different clades. If interspecific gene flow is a rare but taxonomically widespread phenomenon in *Pinus* section *Trifoliae*, then more examples of shared plastid DNA lineages should be found with increased intraspecific sampling.

The assignment of individuals to plastid DNA haplogroups has intrinsic and practical value for providing preliminary species identifications and studying species limits, but even in the absence of introgression, estimates of interspecific variation and the proportion of nonmonophyletic species should increase as intraspecific sampling increases [88]. Consequently, the exclusive reliance on plastid DNA haplotypes for identifying pine species would require us to accept a high error rate. The accuracy of both species identification and delimitation would be greatly improved by the development of additional morphological and genetic markers that can more accurately assess variation and interspecific gene flow.

### Lineage estimation using GMYC

Studies in animals and bacteria have concluded that the GMYC method yields reasonable DNA based species number estimates, and that in animals these are in line with estimates based on morphology [18,19,21,89]. A potential advantage of the GMYC method is that *a priori* assignment of haplotypes to taxa is irrelevant for quantifying the number of species, thus the method may be more suited to analyzing single locus data sets in taxonomic groups where introgression or incomplete lineage sorting are suspected to have occurred.

Given that there is some disagreement in the number of species in *Pinus* section *Trifoliae*, we explored whether a molecular estimate might favor one taxonomic treatment over another. However, over half of the sequences we obtained were identical, no increase was observed in the slope of the lineage-through-time plot, and the GMYC method gave exceptionally low point estimates with broad confidence intervals. In particular, the multimodel point estimates detected 22 or 23 lineages of North American hard pines, or 45-47% of the 49 species that we recognize based on our admitedly more subjective evaluation of morphology and plastid DNA variation. The relaxed molecular clocks gave Miocene crown diversification times for each subsection and the transition time estimates from Yule to coalescent processes were very recent (Pliocene or Pleistocene). The GMYC method has been reported to be less appropriate for recent radiations, and in simulation studies, the method’s accuracy decreased with increasing effective population size [90]. However, the disparity between taxonomic and GMYC estimates found here may be due mainly to low plastid DNA sequence variation and consequently few unique haplotypes per species, thus the application of GMYC with plant plastid DNA may give better estimates as longer sequences are used. Empirical studies are needed to determine whether greater range-wide taxonomic sampling and inclusion of more plastid sequence data will improve the ability of the GMYC methods to recognize more lineages.

## Conclusions

Here we further document the suitability of *ycf1* compared to other commonly used markers for taxonomic identification and phylogenetic reconstruction in closely related pines species, and report an example in which the standard DNA bar code markers for land plants, *matK* and *rbcL*, are inadequate for differentiating among closely related species. Despite several cases consistent with introgression, species-specific clades or paraphyletic grades predominate in subsections *Contortae* and *Australes*. The contrasting pattern of shared haplotypes predominates in subsection *Ponderosae*, which is consistent with relatively recent diversification followed by secondary contact and introgression. For plant groups with low interspecific plastid DNA divergence, successful application of GMYC with plastid DNA may require very long sequences. Finally, for a more complete understanding of phylogeny and species limits, plastid data need to be compared with morphological characters and nuclear or mitochondrial DNA sequences.

## Supporting Information

Appendix S1 Collection information and GenBank Accession numbers for the individuals included in this study. (XLSX)

Appendix S2 Classification of *Pinus* section *Trifoliae* Duhuamel modified from Farjon [28]. (XLSX)

Appendix S3 PCR and sequencing primers used in this study. (XLSX)

Appendix S4 Parsimony tree with gaps included using indel coding. Bootstrap values greater than 50% are shown above branches.
(TIF)

Appendix S5 The best nucleotide substitution models under different partitioning strategies. The likelihood score of the best tree increased with the number of partitions.
(XLSX)

Appendix S6 Chronogram for *Pinus* section *Trifoliae* using two secondary calibration points. Posterior probability values > 0.95 and HPD intervals are indicated on the branches.
(TIF)

Appendix S7 **GMYC results for the tree resulting from the secondary calibration and for the secondary plus fossil calibration.**
(XLSX)
