# Supplementary figures and images for: Phylogenetic Relationships and Species Delimitation in Pinus Section Trifoliae Inferrred from Plastid DNA
Source: PLoS One. 2013 Jul 30;8(7):e70501. doi: 10.1371/journal.pone.0070501 (PMC3728320; doi:10.1371/journal.pone.0070501)

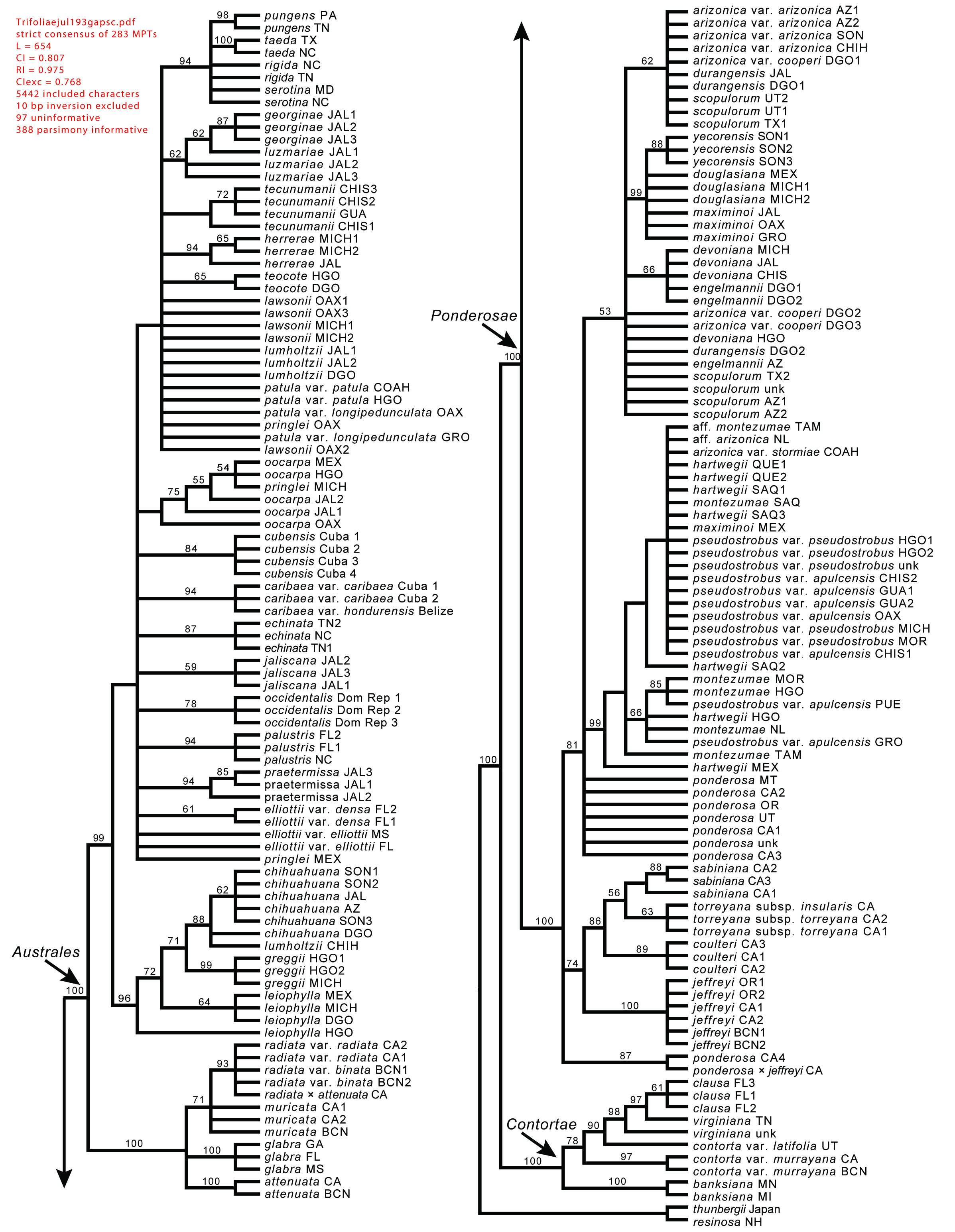

Supplement: Appendix S4 — Bootstrap values greater than 50% are shown above branches. (TIF) [file pone.0070501.s004.tif]

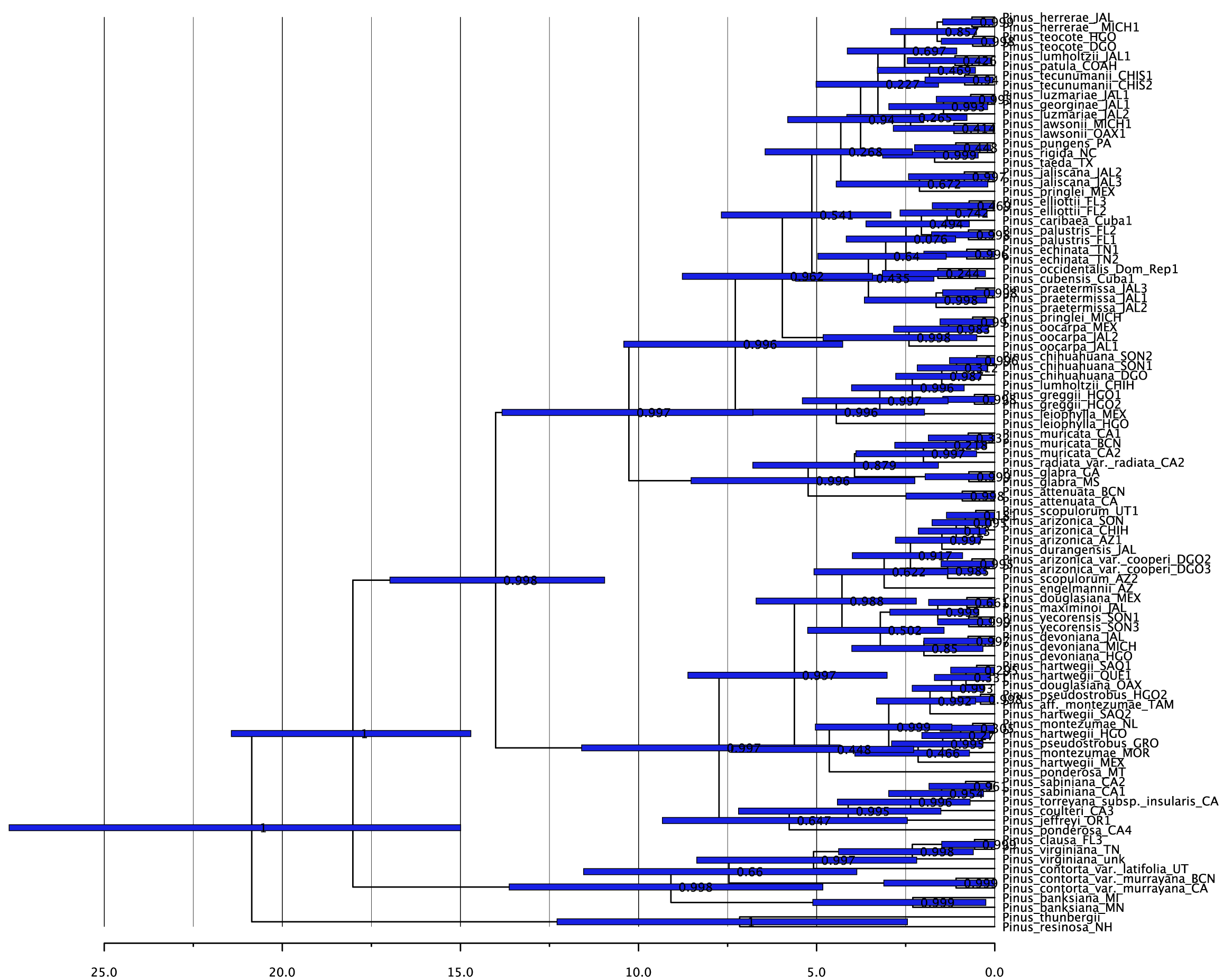

Supplement: Appendix S6 — Posterior probability values > 0.95 and HPD intervals are indicated on the branches. (TIF) [file pone.0070501.s006.tif]
